# The U1 snRNP-specific protein U1C is a key regulator of SMN complex–mediated snRNP formation

**DOI:** 10.1016/j.jbc.2025.110514

**Published:** 2025-07-22

**Authors:** Duc Minh Ngu, Sanat Myti, Ayesha Ali Khan, Jeanne Keita, Tessa Moore, Paul Andega, Alaa Aziz, Ritu Raj, Kayunta Johnson-Winters, Eul Hyun Suh, Byung Ran So

**Affiliations:** 1 Department of Chemistry and Biochemistry, University of Texas at Arlington, Arlington, Texas, USA; 2 Department of Pharmaceutical Sciences, University of North Texas Health Science Center, Fort Worth, Texas, USA

**Keywords:** cancer-associated U1 snRNA mutations, SMN complex, snRNP biogenesis, U1 snRNP, U1C

## Abstract

The stability and abundance of spliceosomal small nuclear ribonucleoproteins (snRNPs) are determined by the assembly of an Sm protein ring (Sm core) on each snRNA, a process orchestrated by the survival of motor neurons (SMN) complex. While the role of the SMN complex as a chaperone is well-established, the mechanisms that regulate its activity remain poorly understood. In this study, we identify U1C, a U1 snRNP-specific protein, as a key regulator of the SMN complex. Using *in vitro* Sm core assembly and protein binding assays, we demonstrate that U1C is essential for Sm core assembly on all snRNAs. In the absence of U1C, Sm core formation on U1 snRNA is disrupted, impairing the SMN complex’s ability to facilitate Sm core assembly on other snRNAs. Furthermore, we show that U1C interacts with the SMN complex *via* post-translational arginine methylations at its C-terminal region, a site distinct from its interaction with U1-70K. Notably, we demonstrate that a prevalent cancer-associated mutation in U1 snRNA, located near the U1C binding site, not only disrupts Sm core assembly but also sequesters the SMN complex, thereby inhibiting canonical snRNP formation. These findings provide important mechanistic insights into how snRNP-specific proteins regulate the SMN complex and suggest that U1 snRNA mutations in numerous cancers may contribute to dysregulation of RNA metabolism by impairing SMN complex activity.

In most eukaryotes, precursor messenger RNAs (pre-mRNAs) transcribed by RNA polymerase II (Pol II) undergo a series of complex and tightly regulated processing steps to produce mRNAs. These steps are crucial for ensuring the correct expression of genes and the production of functional proteins. Nascent pre-mRNAs are co-transcriptionally organized by various RNA-binding proteins and non-coding RNAs (1). One of these most intricate steps in this process is splicing, which involves the removal of introns and the ligation of flanking exons to produce mature mRNAs. This splicing process requires the coordinated association, rearrangement, and dissociation of multiple components of spliceosomes, consisting primarily of small nuclear ribonucleoproteins (snRNPs) and splicing factors (2). In higher organisms, including humans, alternative splicing serves as a key mechanism for generating diverse transcript isoforms from a single gene, thereby fine-tuning protein diversity (3).

Extensive biochemical and structural studies have significantly advanced our understanding of the composition, structure, and stepwise assembly of the spliceosome at the atomic level (4, 5, 6). While the equal stoichiometry of snRNPs required for splicing is widely accepted, recent studies have shown notable variation in the abundance of each snRNP and the snRNP repertoire (snRNPertoire) in a cell- and tissue-specific manner (7, 8). These observations suggest that differential snRNP abundance could play a role in regulating cell-specific gene expression. The mechanisms by which cells maintain distinct snRNP levels and regulate snRNPertoire, however, remain elusive. Moreover, misregulation of splicing—resulting from mutations in splicing factors, snRNP-specific proteins (9, 10), or non-coding snRNAs (11, 12, 13, 14, 15)—can alter splicing outcomes, leading to the production of protein isoforms associated with pathogenesis. Therefore, maintaining a sufficient and diverse repertoire of snRNPs is crucial for ensuring their proper function in spliceosome assembly and splicing, which in turn underpins the regulation of gene expression and the preservation of cellular homeostasis.

All snRNPs contain Sm (or Sm-like) cores, which consist of a heptameric ring of Sm protein surrounding a short RNA sequence known as the Sm site—typically composed of the conserved nucleotide sequence AU_4-6_G. The assembly of the Sm core on each snRNA takes place in the cytoplasm and is chaperoned by the SMN complex (16). The macromolecular SMN complex comprises oligomeric SMN, Gemins2–8, and unrip proteins, which are categorized into three functional subunits (17, 18, 19): 1) SMN/Gemin2 pre-assembles five Sm proteins (D1, D2, E, F, and G) into an intermediate complex essential for Sm protein organization (20, 21, 22, 23); 2) Gemin5/3/4 recognizes the Sm site and the 3′-end of snRNAs, which constitute the snRNP code, ensuring snRNA specificity (18); 3) Gemin6/7/8/unrip bind to the oligomeric SMN protein, which is key to the function of the SMN complex in snRNP biogenesis (24). The Sm site is indispensable for snRNP maturation; when snRNAs lacking Sm sites are overexpressed, they fail to form the Sm core or mature snRNPs and are subsequently degraded by RNA exonucleases (25, 26). This underscores the essential role of the SMN complex in ensuring proper snRNP production and stability. Importantly, SMN protein deficiency causes spinal muscular atrophy (SMA), a neurodegenerative disease affecting motor neurons in infants (27, 28). The severity of SMA is directly correlated with reduced SMN levels, which lead to impaired Sm core assembly (29, 30). Thus, the precise regulation of Sm core assembly by the SMN complex is critical for maintaining the adequate abundance and repertoire of snRNPs within cells.

U1 snRNP is the most abundant spliceosomal snRNP in human cell (*e.g.* HeLa (31)) and plays a pivotal role in regulating co-transcriptional gene expression through two distinct processes in nucleus: splicing and telescripting. In splicing, U1 snRNP recognizes pre-mRNAs through U1 snRNA: 5′ splice site (5'ss) base-pairing during the first step of spliceosome assembly (32). Simultaneously, in telescripting, U1 snRNP suppresses premature cleavage and polyadenylation of nascent transcripts from cryptic polyadenylation signals located in introns and 3′-untranslated regions (33, 34). Furthermore, U1 snRNP determines mRNA length and transcription directionality from bidirectional promoters (35, 36, 37, 38, 39, 40) and interacts with 3′-end termination complexes (41, 42). Recent studies have shown an enrichment of 5’ss motifs in chromatin-associated long non-coding RNAs, suggesting a broader role for U1 snRNP in shaping transcriptome (43). Human U1 snRNP comprises of 11 subunits, including a 5′-trimethyl-G-capped U1 snRNA with four stem-loops (SLs), three U1 snRNP-specific proteins (U1-70K, U1A, and U1C), and a heptameric Sm core (44, 45). Structural evidence of U1 snRNP has provided functional insights into its biogenesis (46, 47, 48). U1 snRNP-specific RNA-binding proteins, U1-70K and U1A, bind SL1 and SL2 of U1 snRNA, respectively. The zinc-finger domain of U1C associates with U1-70K through its N-terminus domain, and the heptameric Sm proteins bind to the Sm site (AUUUGUG) in U1 snRNA. While the SMN complex is essential for snRNP biogenesis, it does not fully explain the relative over-abundance of U1 snRNP. U1-70K, previously known as a component of mature U1 snRNP, hijacks the SMN complex and promotes U1 snRNA-specific Sm core assembly (49). This protein provides a unique pathway within the SMN complex, promoting U1 snRNP formation while suppressing the assembly of other snRNPs.

In this study, we demonstrate that the spliceosomal U1 snRNP-specific protein, U1C, is a key regulator of the SMN complex, playing a crucial role not only in U1 snRNP biogenesis but also in the biogenesis of other snRNPs. Together with U1-70K, U1C facilitates U1 snRNP formation by enabling the SMN complex to recruit other snRNAs:Gemin5 subunit for Sm core assembly. Furthermore, U1 snRNA mutations identified in numerous cancers impair Sm core assembly on U1 snRNA *in vitro*. Notably, a specific point mutation near the U1C binding site impairs the SMN complex’s ability to assemble the Sm core on canonical snRNAs. These findings highlight the pivotal role of U1C in regulating snRNPs biogenesis and suggest that cancer-associated mutations in U1 snRNA may sequester the SMN complex, thereby disrupting broader RNA metabolism.

## Results

### U1 snRNP-specific U1C is essential for the assembly of the Sm core on all snRNAs

**Figure 1:**
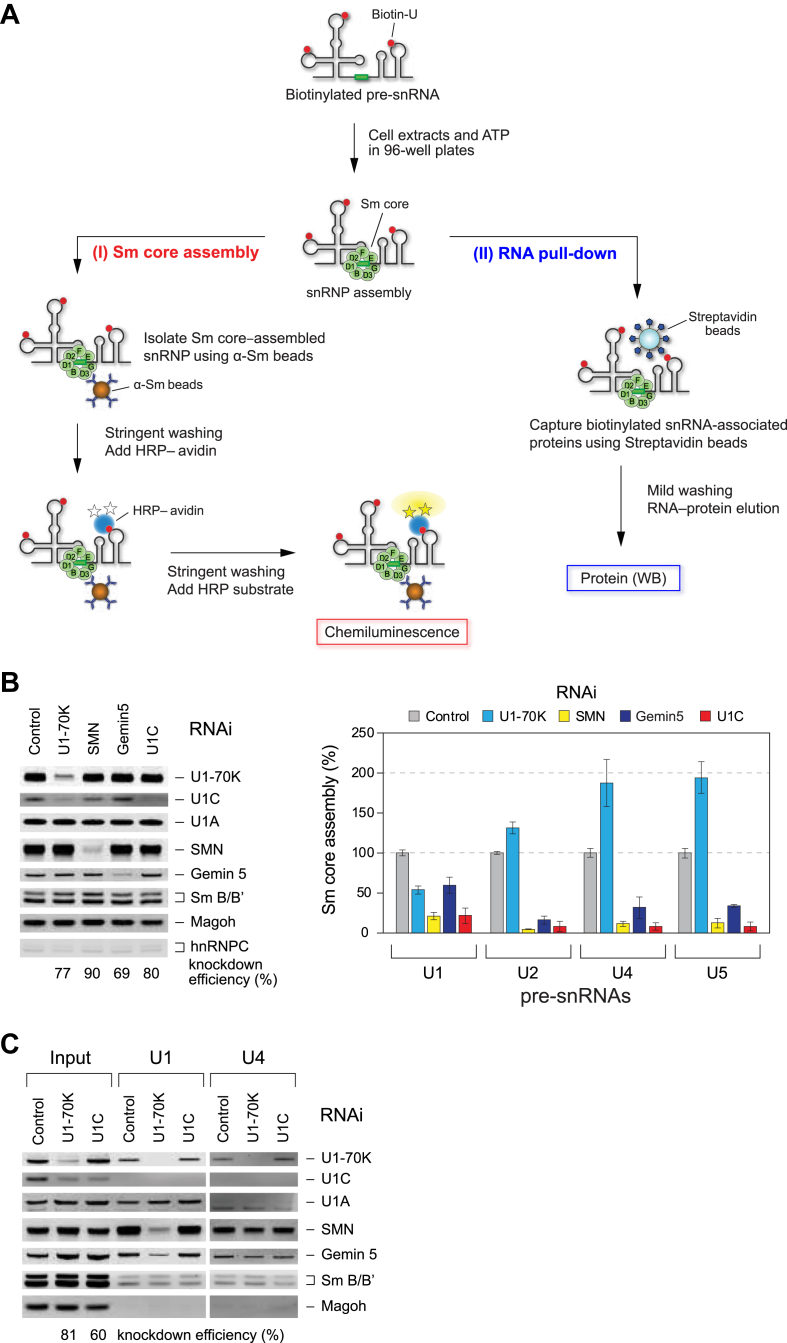
**U1C facilitates Sm core assembly on all spliceosomal snRNAs *in vitro*.***A*, a schematic illustrating the *in vitro* assembly of Sm core and RNA affinity pull-down assays to detect assembled snRNPs and associated proteins. For simplicity, snRNP-specific proteins are not shown. *B*, quantitative Western blot (WB) analysis of the cytoplasmic extracts from HeLa cells with control, U1-70K, SMN, Gemin5, or U1C short interference RNA (siRNA) knockdown. The knockdown efficiencies relative to Magoh protein is shown as a cytoplasmic marker and loading control, with its levels expressed as a percentage relative to the control condition (set at 100%). hnRNPC is included as a nuclear marker to assess the purity of the cytoplasmic extracts. The Sm core assembly activities on each snRNA in the same cell extracts are compared with control RNAi extracts (100% activity). Error bars represent the standard deviation (SD) from three independent cell cultures. *C*, WB analysis of SMN complex proteins bound to biotinylated pre-U1 and pre-U4 snRNAs in HeLa cells with control, U1-70K, or U1C siRNA knockdown in the presence of ATP. The input lanes show 20% of each of the cell extracts used. The knockdown efficiencies relative to Magoh protein are indicated.

U1-70K serves as a bridge between U1 snRNA and the SMN complex, providing an additional and U1 snRNP-specific pathway for Sm core assembly (49). Interestingly, knockdown of U1-70K protein leads to a concomitant reduction in U1C protein expression, whereas the reverse is not observed (49, 50, 51), suggesting an interdependent relationship between these proteins. To investigate the role of U1C in snRNP biogenesis, we utilized an *in vitro* Sm core assembly assay, a high-throughput method for quantifying Sm core-assembled snRNAs using an anti-Sm antibody (Fig. 1*A*, Sm core assembly). In this assay, *in vitro* transcribed and biotinylated precursor U1 snRNA (pre-U1), along with other snRNAs (pre-U2, pre-U4, and pre-U5), or a negative control (an Sm site mutant, ΔSm (16)), were incubated in cytoplasmic extracts, respectively. Subsequently, ATP-dependent Sm core-assembled snRNAs were enriched using anti-Sm antibody-immobilized magnetic beads in a 96-well format. The immunopurified snRNA-protein complexes were then incubated with horseradish peroxidase-conjugated avidin, and the associated snRNAs were quantified by chemiluminescence.

HeLa cell extracts were first used in which U1 snRNP-specific proteins (U1-70K and U1C) and SMN complex proteins (SMN and Gemin5) had been knocked down by RNA interference (RNAi). Compared to control, target-specific siRNAs resulted in a 70 to 90% reduction in protein levels in the cytoplasmic fraction, as confirmed by Western blot (WB) (Fig. 1*B*). As expected, knockdown of U1-70K resulted in a 50% reduction in Sm core assembly activity for U1 snRNA, whereas assembly on other snRNAs showed a 30% to 100% increase (Figs. 1*B*, S1). Gemin5 knockdown significantly decreased Sm core assembly on other snRNAs by 65 to 85%, while reducing assembly on U1 snRNA by less than 40%. SMN knockdown completely abolished Sm core assembly on all snRNAs, underscoring its pivotal role in this process. Strikingly, U1C knockdown significantly reduced Sm core assembly not only on U1 but also on other snRNAs, similar to the effect of SMN knockdown. Overexpression of U1 snRNP-specific proteins in HEK293T cells differentially affected Sm core assembly on all snRNAs *in vitro* (Fig. S2). In contrast to U1-70K knockdown by siRNA, overexpression of U1-70K alone did not alter U1C expression levels, and *vice versa*. U1-70K and U1A primarily enhanced Sm core assembly on U1 snRNA, whereas U1C did not. Notably, overexpression of both U1-70K and U1C increased Sm core assembly on other snRNAs, indicating that these proteins independently contribute to U1 snRNP biogenesis and may also influence the assembly of other snRNPs. These findings suggest a broader regulatory role for U1C in Sm core assembly across spliceosomal snRNAs, extending beyond its canonical function as a U1 snRNP-specific component.

### U1C coordinates SMN complex interaction and regulates broad snRNP biogenesis

To examine whether U1C influences the interaction between snRNAs and the SMN complex, we performed *in vitro* snRNA affinity pull-down experiments (Fig. 1*A*, RNA affinity pull-down). The binding assays were carried out at 30 °C in the presence of ATP to capture intermediate complexes of SMN complex and snRNP-specific proteins on biotinylated snRNAs during Sm core assembly. HeLa cells were treated with specific siRNAs to knock down target proteins, and cytoplasmic extracts were incubated with pre-U1 and pre-U4 snRNAs. The resulting snRNA-protein complexes were then isolated using streptavidin beads and analyzed by WB (Fig. 1*C*). Notably, U1-70K knockdown abolished U1 snRNA binding to the SMN complex, while U4 snRNA binding remained unaffected. In contrast, U1C knockdown maintained the binding of both U1 and U4 snRNAs to the SMN complex compared to the control knockdown. Under mild washing conditions (200 mM NaCl and 0.2% Triton X-100), U1C did not tightly bind to U1 snRNA in the control, likely because it lacks an RNA-binding domain and instead associates with the Sm core *via* the N-terminus of U1-70K (52, 53). Strikingly, the U1C knockdown did not disrupt the interaction between the SMN complex and pre-U1 or pre-U4 snRNA, although it completely prevented Sm core assembly. Notably, pre-U4 snRNA is associated with U1-70K, not U1A, suggesting that the interaction is not snRNA-mediated, but rather occurs through the oligomeric SMN associated with U1-70K (45).

**Figure 2:**
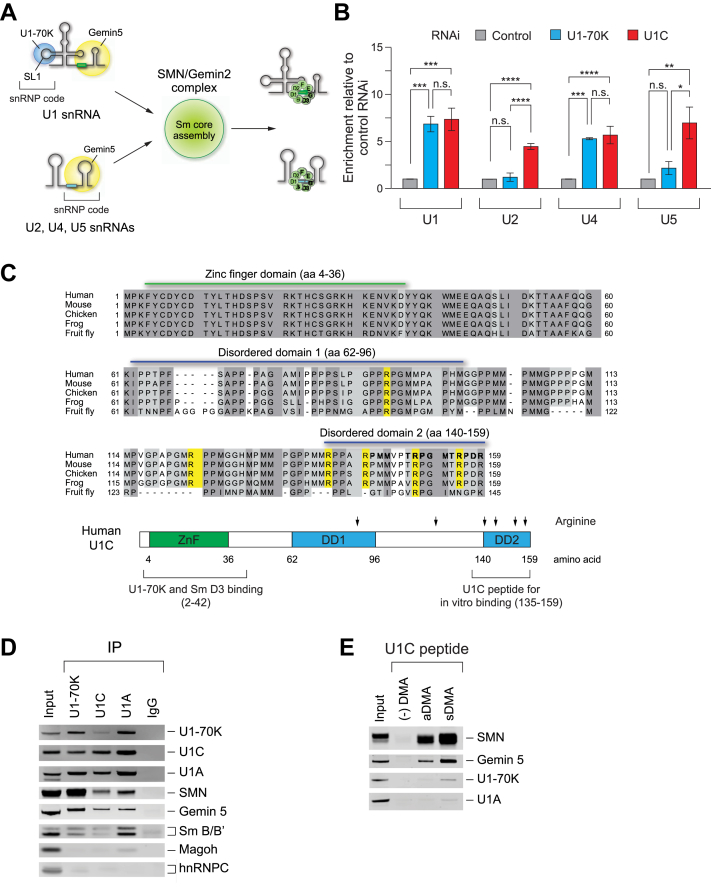
**U1C regulates the association of snRNAs with Gemin5 and in cell abundance of snRNP abundance through its the interaction with the SMN protein.***A*, a schematic representation of snRNA binding model with U1-70K and/or Gemin5 for Sm core assembly, facilitated by the SMN/Gemin2 complex. U1 snRNA interacts with U1-70K through stem loop (SL) one and with Gemin5, both of which are associated with the SMN/Gemin2 complex. Other snRNAs (*e.g.* U2, U4, and U5) are recognized by Gemin5 and similarly recruited to the SMN/Gemin2 complex. The snRNP code for interaction with the SMN complex within each snRNA is indicated. *B*, quantitative measurement of snRNAs associate with Gemin5 in HeLa cells with control, U1-70K, or U1C siRNA knockdown by quantitative real-time PCR (qRT–PCR). Immunoprecipitations were performed using anti-Gemin5 in cells crosslinked with 0.2% formaldehyde. The relative amounts of each snRNAs compared with control knockdown set are shown. Error bars indicate the SEM from three biological replicates. Statistical significance was determined by one-way ANOVA with Bonferroni *post hoc* test (∗*p* < 0.05, ∗∗*p* < 0.01, ∗∗∗*p* < 0.001, ∗∗∗∗*p* < 0.0001; n.s., not significant). *C*, sequence alignment of U1C protein sequences from human (*Homo sapiens*), mouse (*Mus musculus*), chicken (*Gallus gallus*), zebrafish (*Danio rerio*), and fruit fly (*Drosophila melanogaster*), performed using the Praline program. Partially conserved residues (conservation score 4–7) are highlighted in *light gray*, while highly conserved residues (conservation score 8–10) are highlighted in *dark gray*. U1C’s secondary structure is indicated by a *green line* for the zinc finger (ZnF) domain and *blue lines* for the disordered domain (DD). The positions of arginine residues predicted for methylation using the PRmePRed program are highlighted in *yellow*. A schematic diagram illustrates the human U1C protein domain organization, the positions of arginine, and the U1-70K and Sm D3 binding domain (aa 2–42). Additionally, the U1C peptides (aa 135–159) used for *in vitro* binding assays are indicated. *D*, WB analysis of immunoprecipitated U1 snRNP-specific proteins from cytoplasmic HeLa cell extracts using anti-U1-70K, anti-U1C, anti-U1A antibodies, and mouse immunoglobulin G (IgG). The associated U1 snRNP-specific proteins, SMN, Gemin5, and Sm B/B′ are indicated. The input *panel* shows 2.5% of the proteins used for binding. *E*, *In vitro* binding assays were performed using biotinylated U1C C-terminus domain peptides with or without di-methylated arginine (DMA) in cytoplasmic HeLa extracts. The U1C peptides included those without di-methylarginine (-DMA), asymmetrically di-methylated arginine (aDMA), and symmetrically di-methylated arginine (sDMA) at positions 140, 144, 151, and 156. The input *panel* shows 2.6% of the total proteins used.

U1 snRNA interacts with the SMN complex *via* two RNA binding proteins: U1-70K, which binds to SL1, and Gemin5, which recognizes SL four and the Sm site (49, 54, 55). In contrast, other snRNAs depend exclusively on Gemin5 for their association with the SMN complex (56). Gemin5 subsequently delivers these snRNAs to the SMN/Gemin2 subunit for Sm core formation (18, 20, 45) (Fig. 2*A*). To further investigate whether U1 snRNP-specific proteins influence Gemin5 and snRNA interactions, we performed immunoprecipitation experiments to assess the effects of U1-70K or U1C knockdown on Gemin5’s association with snRNAs. To capture transient snRNAs-Gemin5 interactions, HeLa cells transfected with U1-70K or U1C RNAi were crosslinked using 0.2% formaldehyde prior to anti-Gemin5 immunoprecipitation. This mild in-cell crosslinking preserves labile complexes by minimizing reassociation or dissociation during stringent wash steps following cell lysis. After RNA–protein complex elution and reversal of crosslinking, snRNA abundance was analyzed using real-time quantitative PCR (RT–qPCR) (Fig. 2*B*). U1-70K knockdown increased U1 and U4 snRNA binding with Gemin5 by 4- to 6-fold compared to control knockdown. In contrast, U2 snRNA binding remained unchanged, while U5 snRNA binding increased by 2-fold. Notably, U1C knockdown increased binding of all tested snRNAs to Gemin5 by 4- to 7-fold compared to control knockdown. These results indicate that, despite U1C and U1-70K being the key components of U1 snRNP, these two proteins regulate distinct snRNAs association with Gemin5. The differential bindings of snRNAs to Gemin5 also support the previous observation that at least two distinct snRNA binding sites exist within the SMN complex: one with high affinity for U1 and U4 snRNAs, and another with reduced affinity for U2 and U5 snRNAs (56). Thus, these findings demonstrate that U1C plays a critical role in regulating the access of snRNAs-Gemin5 to the SMN complex for Sm core assembly.

### U1C interacts with SMN and U1-70K *via* its two distinct domains

We hypothesized that upon U1C knockdown (Fig. 1*B*), the reduced Sm core assembly *in vitro* could result from a disruption in the interaction between U1C and the SMN complex. Biochemical and structural studies on U1 snRNP support the function of U1C in stabilizing the mature U1 snRNP through interactions of its N-terminal domain (NTD) with U1-70K and Sm core proteins (45, 46, 47, 54). However, the role of U1C’s C-terminal domain (CTD) in snRNP biogenesis remains unclear, as it is not visible in the human U1 snRNP structure. Sequence alignment of U1C orthologs from diverse organisms reveals a highly conserved zinc finger (ZnF) domain in the NTD, which interacts with U1-70K and and Sm D3, as well as disordered domains (DD) in the CTD (Fig. 2*C*). Within the CTD, proline–glycine–methionine (PGM) motifs are post-translationally modified to di-methylated arginines (DMAs) by CARM1/PRMT4 or PRMT5 methyltransferases (57). To test whether U1C directly interacts with the SMN complex, we performed immunoprecipitation experiments in cytoplasmic HeLa cell extracts using anti-U1-70K, anti-U1C, and anti-U1A antibodies. Subsequent WB analysis revealed that all U1 snRNP-specific proteins bind to SMN proteins, with U1-70K exhibiting the strongest interaction, followed by U1A and U1C (Fig. 2*D*). In parallel, co-immunnoprecipitation with anti-SMN and anti-Gemin5 antibodies confirmed stable interaction with U1-70K and U1A, but not U1C (Fig. S3*A*). To evaluate whether these interactions are RNA-dependent, we performed RNase A/T1 treatment prior to immunoprecipitation (Fig. S3*B*). U1-70K remained associated with the SMN complex following RNase treatment, consistent with previous studies (58) that demonstrate RNA-independent binding. In contrast, U1C showed a moderate reduction in its association with SMN and Gemin5 after RNase treatment, suggesting that its interaction is at least partially dependent on the presence of U1 snRNA.

Next, to validate whether DMA modifications of U1C enhance its interaction with SMN protein, we designed synthetic 25-mer peptides containing the PGM motifs of U1C CTD (aa 135–159) with a biotin tag at the N-terminus (Fig. 2*C*, Table S1). These peptides were designed with symmetrically (sDMA) or asymmetrically (aDMA) dimethylated arginines, or with no modifications at all. We performed *in vitro* binding assays using these biotinylated U1C peptides, incubating them in cytoplasmic HeLa cell extracts. The associated proteins were then eluted and analyzed by WB (Fig. 2*E*). We observed a robust association between SMN and the sDMA- or aDMA-modified U1C peptide, with a preference for sDMA. Other components of the SMN complex (*e.g.* Gemin5) were also associated with U1C. However, U1-70K and U1A did not interact with U1C CTD. Notably, the unmodified U1C peptide did not bind the SMN complex. These results support that DMA modifications of U1C enhance its interaction with the SMN protein, which contains a Tudor domain known to bind proteins with methylated arginine or lysine residues (59, 60, 61). Moreover, our findings suggest that U1C’s dual binding to U1-70K *via* its NTD and to SMN *via* its CTD is essential for U1 snRNP formation.

### Mutations in U1 snRNA identified in various cancers disrupt Sm core assembly on snRNAs and hinder the formation of snRNPs

**Figure 3:**
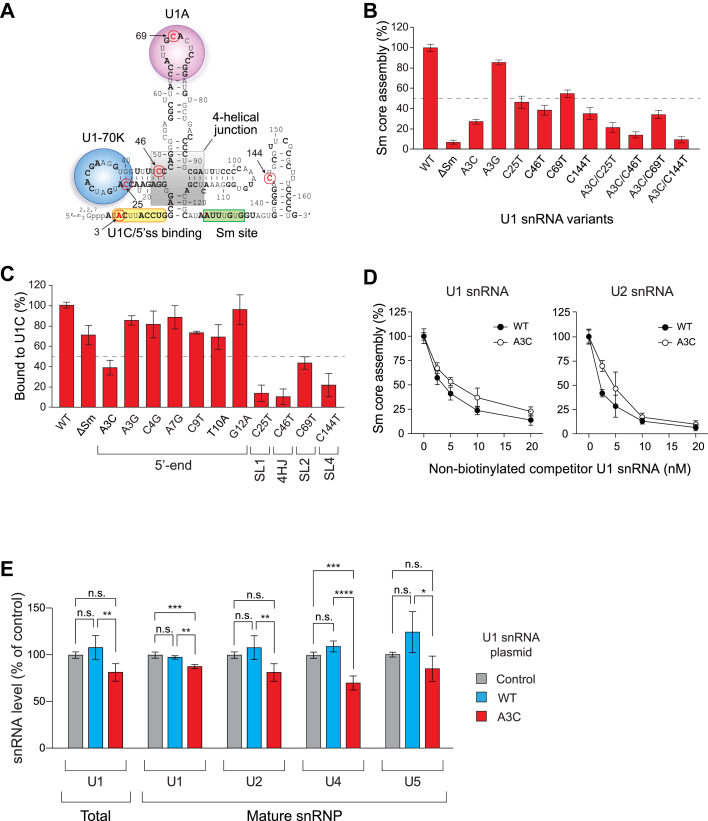
**U1 snRNAs mutations identified in cancers disrupt Sm core assembly *in vitro* and alter snRNP abundance in cells.***A*, secondary structure of U1 snRNA, with binding sites for U1-70K, U1A, and U1C/5'ss indicated by *circles*. The Sm site and 4-way helical junction are highlighted in *boxes*. Nucleotide residues mutated in pan-cancer patient samples are shown in bold, and residues analyzed in this study are marked with *arrows*. *B*, the Sm core assembly activities on U1 snRNA mutants (A3C, C25T, C46T, C69T, C144T) compared to WT, set at 100%. ΔSm serves as a negative control. *C*, *in vitro* U1C binding assays with U1 snRNA mutations at the 5′-end, stem loops (SL1, SL2, and SL4), and a 4-way helical junction (4HJ). U1C-associated snRNAs were immunoprecipitated using U1C-specific antibodies. *D*, A3C U1 snRNA inhibits Sm core assembly on WT U1 and U2 snRNAs in a dose-dependent manner. *In vitro* Sm core assembly assays were performed using 10 nM biotinylated WT U1 or U2 snRNA in the presence of increasing concentrations (2.5–20 nM) of non-biotinylated WT (*filled circles*) or A3C (*open circles*) U1 snRNA, respectively. Data are shown as percentages relative to Sm core assembly activity in the absence of competitor RNA (set to 100%). *E*, quantitative analysis of snRNAs from total RNA and mature snRNPs in HEK293T cells transfected with WT, A3C U1 snRNA, or a control plasmid. Mature snRNPs were immunopurified using an anti-Sm antibody, and snRNA levels were measured by qRT–PCR. Data are shown as percentages relative to control plasmid-transfected cells (set to 100%). *Error bars* in *panels (B–D)* represent the SD from three biological replicates. *Error bars* in *panel (E)* indicate the SEM from three biological replicates. Statistical significance was determined by one-way ANOVA with Bonferroni *post hoc* test (∗*p* < 0.05, ∗∗*p* < 0.01, ∗∗∗*p* < 0.001, ∗∗∗∗*p* < 0.0001; n.s., not significant).

Somatic mutations in U1 snRNA across multiple cancer types suggest potential alterations in pre-mRNA splicing (11, 12). Interestingly, these mutations were distributed throughout the U1 snRNAs transcript, raising concerns about the stability and function of U1 snRNP containing these mutations (Fig. 3*A*). To investigate the impact of U1 snRNA mutations on Sm core assembly and snRNP formation, we selected several mutations previously reported in 240 patients with cancer, spanning 30 different cancer types (12). The 3A > C and 3A > G mutations in U1 snRNA were our primary targets, as they are the most frequent mutations found in medulloblastoma, chronic lymphocytic leukemia (CLL), and hepatocellular carcinoma (HCC). These mutations are located at the 5′-end of U1 snRNA, a region critical for 5′ splice site recognition (32). Interestingly, the A3C mutation resulted in a 70% reduction in Sm core assembly *in vitro* compared to the wild-type (WT), while the A3G mutation had only a minor effect, reducing assembly by 13% (Fig. 3*B*). In addition, mutations in other SLs or 4-way helical junction (4HJ), such as C25T (SL1), C69T (SL2), C46T (4HJ), and C144T (SL4), which are binding sites for U1-70K, U1A, and SF3A1 (62) or are crucial for U1 snRNA structure (63), reduced Sm core assembly by 40 to 60%. As expected, the ΔSm mutation, which replaces AUUUGUG to CUCGAG, completely abolished Sm core formation. Moreover, double mutations involving A3C and other SL or 4HJ regions, identified in some CLL, B-cell non-Hodgkin lymphoma (B-NHL), and HCC patients, led to more severe defects in Sm core assembly compared to single mutations (Fig. 3*B*). Further analysis of additional 5′-end U1 snRNA mutations (C4G, A7G, C9T, T10A, and G12A) revealed similar, albeit less severe, reductions in Sm core assembly (20–50%) (Fig. S4). Given the proximity of U1C to the 5′-end of U1 snRNA in the mature U1 snRNP, we performed *in vitro* Sm core assembly assays using anti-U1C antibodies and observed a correlation between Sm core formation and U1C association (Fig. 3*C*). Among the tested 5′-end U1 snRNA mutations, A3C exhibited a 60% reduction in U1C binding, whereas other residues showed only minor defects (5–27%). Notably, the C25T mutation in SL1, the binding site for U1-70K, significantly reduced U1C association during Sm core assembly (87%). Mutations in SL2, SL4, and 4HJ also showed that U1C association with these mutants correlates with Sm core assembly *in vitro* (Fig. 3, *B* and *C*). These findings are consistent with previous studies showing that the U1-70K: Sm core complex creates a binding site for U1C (45, 64). These results further suggest that U1-70K binding to U1 snRNA is a prerequisite for U1C association during Sm core assembly.

Since both the SMN complex and U1-70K recognize U1 snRNA, we hypothesized that the A3C U1 snRNA mutant competes for shared binding sites but fails to associate with U1C, which is essential for U1 snRNP formation. To access the impact of A3C on Sm core assembly of U1 and other snRNAs *in vitro*, we performed competition-based Sm core assembly assays. Biotinylated WT U1 snRNA was incubated with cytoplasmic extracts in the presence of increasing concentrations (2.5–20 nM) of non-biotinylated WT or A3C U1 snRNA. Both WT and A3C U1 snRNA competitors similarly reduce Sm core assembly on pre-U1 snRNA (Fig. 3*D*). Notably, at equivalent concentration (*e.g.*, 10 nM of biotinylated snRNA and competitor RNAs), both competitors more effectively inhibited Sm core assembly on U2 snRNA than on U1. To further investigate whether U1 snRNA mutations affect interactions with the SMN complex and U1 snRNP-specific proteins, we performed RNA affinity pulldown assays using biotinylated mutant snRNAs (Fig. S5). The A3C mutant maintained associations with SMN, Gemin5, and U1-70K, indicating that this single nucleotide change does not significantly disrupt these interactions. Likewise, the C25T mutation in SL1 results in only a modest reduction in U1-70K binding. The ΔSm mutant binds Gemin5, likely due to the presence of two binding sites: SL1 (*via* U1-70K) and SL4/Sm site (*via* Gemin5) (56, 64). These results suggest that mutant U1 snRNAs may sequester the SMN complex by maintaining partial or attenuated interactions with limiting assembly factors, thereby interfering with efficient Sm core assembly.

To assess the impact of A3C expression on mature snRNP levels in cells, we measured total U1 snRNA in HEK293T cells transfected with plasmids expressing WT or A3C U1 snRNA, driven by the endogenous promoter and terminator sequences, or a control plasmid (Fig. 3*E*). Additionally, mature snRNPs were isolated from these cells using anti-Sm antibodies, and snRNA abundance was quantified by qRT–PCR. WT U1 snRNA expression led to similar or slightly elevated levels of U1 snRNA and snRNPs, whereas A3C expression gradually reduced the abundance of U1 and other snRNPs up to 30%. Together, these *in vitro* and in cells results suggest that cancer-associated U1 snRNA mutations disrupt Sm core assembly by impairing the association of U1 snRNP-specific proteins or by altering U1 snRNA structure. Specifically, the A3C mutation, one of the most frequently observed in cancer, binds overlapping sites on the SMN complex and U1-70K, but not U1C, thereby inhibiting Sm core assembly of both U1 and other snRNAs. Consequently, A3C may sequester the SMN complex, hindering the formation of canonical snRNPs.

## Discussion

**Figure 4:**
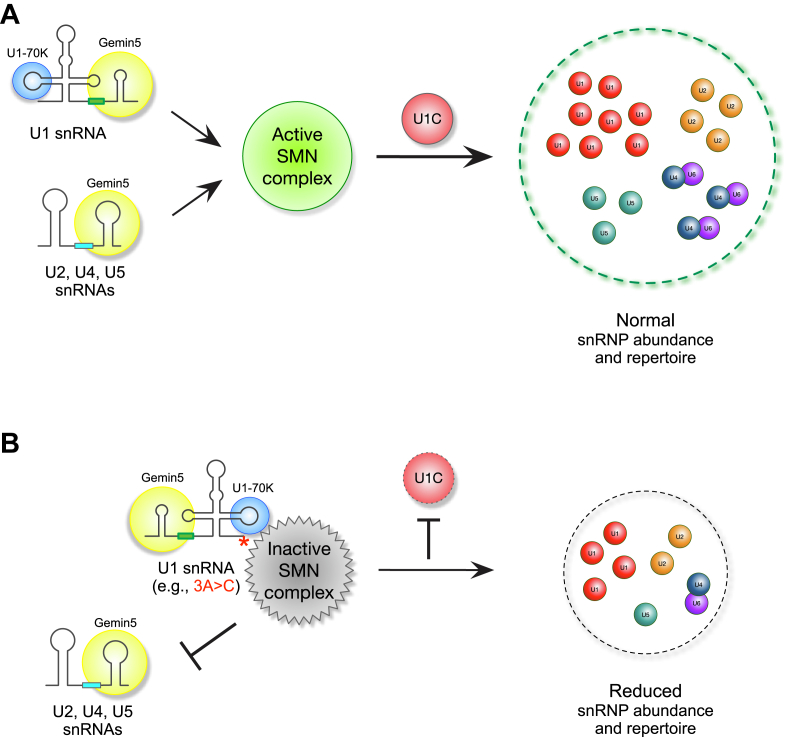
**Schematic representation of U1 snRNP-specific U1C’s role as a gatekeeper of the SMN complex, regulating Sm core assembly for all snRNAs.***A*, U1C associates with a U1 snRNP intermediate, comprising U1-70K and U1 snRNA, within the SMN complex to form U1 snRNP. U1C-assisted U1 snRNP formation enables the SMN complex to recruit Gemin5 and other snRNAs (U2, U4, or U5) and produce other snRNPs. In the absence of U1C, the SMN complex bound to the U1-70K:U1 snRNA intermediate fails to produce U1 snRNP and blocks access of other snRNA:Gemin5 subunits to the SMN complex. *B*, cancer-relevant mutations in the 5′ end of U1 snRNA (*e.g.*, 3A > C) abolishes U1C binding, sequestering the SMN complex with the U1-70K:U1 snRNA mutant and thereby disrupting Sm core assembly on both canonical U1 snRNA and other snRNAs. The point mutation is marked by an *asterisk*.

The multi-component SMN complex plays a crucial role in snRNP biogenesis by facilitating Sm core formation on snRNAs (17, 65). However, the mechanisms regulating its chaperone activity remain an open question. While U1-70K and U1C were previously recognized as components of mature U1 snRNP, our study uncovers broader and distinct roles for U1C in snRNP biogenesis. Together with U1-70K, U1C facilitates the formation of both U1 and other snRNPs by utilizing the SMN complex in a substrate-assisted manner. Based on the U1 snRNP structures (46, 47, 48), our biochemical data suggest a stepwise formation of U1 and other snRNPs, mediated by the SMN complex. U1-70K promotes the assembly of Sm core on U1 snRNA by bridging between U1 snRNA and the SMN complex through its RNA binding domain (49). This preferential interaction between U1-70K:U1 snRNA and SMN protein, in turn, limits the access of other snRNAs to the SMN complex, thereby suppresses Sm core assembly and explaining the relative overabundance of U1 snRNP. Pre-assembled U1 snRNP, comprising U1-70K:U1 snRNA and the Sm core, provides an additional interaction site for U1C’s NTD, which helps stabilize the mature U1 snRNP. U1C further associates with the SMN protein through its CTD *via* post-translational arginine modifications, enhancing the interaction with Tudor domain of SMN. In the absence of U1C, the pre-assembled U1-70K:U1 snRNA is unable to dissociate from the SMN complex. This disruption prevents the SMN complex from recruiting other snRNAs through Gemin5, ultimately inhibiting formation of other snRNPs. Taken together, the findings from our study suggest that U1C plays dual roles in snRNP biogenesis as a ‘gatekeeper’ of the SMN complex: 1) U1C facilitates the completion of mature U1 snRNP formation, and 2) it enables the SMN complex to recruit other snRNAs *via* Gemin5 and promotes snRNP assembly (Fig. 4*A*). In addition, post-translational modifications, such as phosphorylation and arginine methylation, are known to regulate the association of SMN complex subunits (66, 67, 68), potentially contributing to the dissociation of mature snRNPs from the SMN complex and enhancing the snRNA recruitment for Sm core assembly.

We also observed that the apparent discrepancy between U1-70K knockdown and overexpression arises from the co-depletion of U1C protein that accompanies U1-70K knockdown, as previously reported (49, 50, 51). Structural studies of the human U1 snRNP further support this interdependence, demonstrating a direct protein–protein interaction between U1-70K and U1C (46, 47, 48). Our data suggest that U1C and U1-70K contribute independently to U1 snRNP biogenesis and may also influence the assembly of other snRNPs, potentially by modulating SMN complex activity or by affecting snRNA specificity *via* Gemin5.

The SMN complex, a well-established RNP-exchange depot, associates with diverse RNA-binding proteins, including like-Sm proteins, snoRNA-binding proteins, and fibrillarin, *via* its Tudor domain (68, 69, 70, 71). Importantly, the SMN complex exists in heterogeneous, higher-order oligomeric forms (72). It is plausible that distinct SMN-containing subcomplexes play different roles during snRNP biogenesis and other RNA processing events. Our current experimental design does not differentiate among these oligomeric or modified SMN states, which may complicate the interpretation of the observed binding dynamics in immunoprecipitation assays. While U1C appears to influence snRNP assembly dynamics, its function likely extends beyond a single mechanistic step, such as facilitating release *versus* stabilization of the snRNP and may depend on the context or composition of the SMN subcomplexes involved. Further studies will be required to dissect these mechanistic details and the functional relevance of distinct SMN assemblies. Nonetheless, our findings underscore the broader significance of the SMN complex beyond its canonical role in snRNP biogenesis and its disease-relevant link, such as SMA. For instance, cancer-associated mutations in U1 snRNAs may sequester SMN complex chaperones, thereby perturbing snRNP homeostasis (Fig. 4*B*). Moreover, the association of U2 and U4 snRNAs or Gemin5 mutations with developmental and neurodegenerative disorders further supports the expanded roles of the SMN complex (14, 15, 73, 74).

Frequent mutations in splicing factors or dysregulation of pre-mRNAs have been implicated in cancer pathogenesis (9, 10, 75). Recent studies have revealed that non-coding RNAs, including snRNAs, tRNAs, and long non-coding RNAs, play crucial roles in cancer development and progression (76, 77, 78, 79). Based on our findings, we propose that mutations in U1 snRNA disrupt snRNP biogenesis homeostasis, contributing to cancer development. Our data demonstrate that the 5′-end of U1 snRNA is essential for Sm core assembly, and cancer-associated mutations in this region offer a valuable model for investigating the physiological roles of U1 snRNA in cancer biology. Although these mutations occur at low frequency in patients with cancer, even a small fraction of U1 snRNA mutants could exert significant effects. These mutations may impair U1 snRNP formation and/or sequester the SMN complex, leading to compromised biogenesis of not only U1 but also other snRNPs. Furthermore, the differential abundance of snRNAs (7, 8, 31) may contribute to their variable sensitivity to changes in SMN protein availability. This disruption, in turn, could impede proper spliceosomes assembly, creating an imbalance between available snRNPs and the demands of transcription, pre-mRNA processing, or translation (34, 80, 81). Notably, U1 snRNA mutations such as the A3>G mutation, have been identified in Sonic Hedgehog Medulloblastoma, while A3>C mutation is predominantly found in CLL and HCC (11, 12). Although the precise mechanisms by which these mutations differentially affect Sm core assembly and splicing remain unclear, atomic-level structural information of U1 snRNA carrying the mutations could provide valuable insights into its impact on U1 snRNP formation and alternative splicing.

## Experimental procedures

### Cell culture, transfection, and formaldehyde crosslinking

HeLa and HEK293T cells were cultured in DMEM supplemented with 10% (v/v) fetal bovine serum (FBS), 2 mM L-glutamine, 10 units/ml penicillin, and 10 μg/ml streptomycin at 37 °C with 5% CO_2_. HeLa cells were transfected with control siRNA or siRNA targeting U1C, U1-70K, SMN, and Gemin5 (Dharmacon, Horizon Discovery) using RNAiMax (Invitrogen). After 42 to 46 h of incubation, cells were harvested for snRNA measurements, *in vitro* Sm core assembly, or RNA affinity pull-down assays.

U1 snRNAs in pBlueScript plasmid were transfected in HEK293T cells using the NEON transfection system (Invitrogen) *via* electroporation of 5 × 10^6^ cells with two pulses of 20 ms each at 1100 V. Following electroporation, cells were incubated in DMEM in a 15-cm plate. After 42 to 48 h of incubation, cells were harvested and washed twice with ice-cold PBS. To capture Gemin5-associated snRNAs in cells, HeLa cells were fixed with 0.2% (w/v) formaldehyde for 10 min at room temperature with gentle rotation. The reactions were quenched with glycine (pH 7.0) to a final concentration of 150 mM for 10 min, followed by two washes with ice-cold PBS.

### U1C peptides

Arginine methylation sites in the U1C C-terminus domain were identified using the Protein arginine methylation prediction tool (PRmePRed (82), http://bioinfo.icgeb.res.in/PRmePRed/). Custom-designed N-terminus biotinylated U1C peptides were obtained from Sigma–Aldrich. Each peptide was dissolved in UltraPure water (Invitrogen) to prepare a stock solution at 2 mM and further diluted to 20 μM in a buffer containing 10 mM Tris-HCl (pH 7.5), 50 mM NaCl for *in vitro* binding studies. Details of all peptide information are provided in Table S1.

### Construction of precursor snRNAs and *in vitro* transcription

Precursor U2, U4, and U5 snRNAs were amplified from HeLa cell genomic DNA using Phusion Plus PCR Master Mix (ThermoScientific) and subsequently inserted into the pUC19 plasmid between EcoRI and HindIII. Mutations in precursor U1 snRNA were introduced *via* QuikChange Lightning Site-Directed Mutagenesis Kit (Agilent). All snRNA sequences were verified by Sanger Sequencing (Eurofinsgenetics). Linearized pre-snRNA plasmids with BsaI digestion were used for *in vitro* transcription using MEGAscript T7 Transcription Kit (Invitrogen) in the presence of 1.75 mM biotin-16-UTP (Roche) and 1.875 mM UTP. Transcribed RNAs were purified by electrophoresis on a 6% polyacrylamide gel containing 5 M urea; followed by phenol:chloroform extraction, ethanol precipitation, and resuspension in RNase-free DEPC water. The concentrations of pre-snRNAs were determined by UV absorbance at 260 nm. Details of all primer sequences are provided in Table S2.

### Preparation of HeLa cytoplasmic extracts

HeLa S3 cells were resuspended in reconstitution buffer [20 mM HEPES/KOH pH 7.9, 50 mM KCl, 5 mM MgCl_2_, 0.2 mM EDTA] containing 100 μg/ml digitonin and protease inhibitor, then passed through a 25G needle five times on ice. The cell lysate was clarified by centrifugation at 1500*g* for 1 min, and the supernatant was mixed with NP-40 to a final concentration of 0.01%. After a second centrifugation at 9400*g* for 15 min at 4 °C, the supernatant was collected, glycerol was added to a final concentration of 5%, and the mixture was stored at −80 °C. The concentration of various extracts was determined by the Bradford protein assay (Pierce).

### Antibodies, immunoprecipitation, and quantitative immunoblotting

The following antibodies were used for this study: anti-SMN (BD Bioscience or 2B1), anti-Gemin5 (Proteintech), anti-Sm (Sigma–Aldrich), anti-U1C (Sigma–Aldrich), anti-U1-70K (Synaptic Systems or Santa Cruz Biotechnology), anti-U1A (Proteintech), and anti-Magoh (Santa Cruz Biotechnology).

Immunoprecipitation was performed using anti-U1C, anti-U1-70K, anti-SMN, and anti-Gemin5 antibodies immobilized on Dynabeads Protein G (Invitrogen). The beads were incubated with cytoplasmic extracts (2 mg/ml) in RSB-100 buffer [10 mM Tris-HCl (pH 7.5), 100 mM NaCl, 2.5 mM MgCl_2_] containing 0.1% NP-40 for 1 h at 4 °C in a 96-well plate. After incubation, the beads were washed five times with RSB-200 buffer containing 0.02% NP-40 using a KingFisher Flex magnetic beads processor (ThermoFisher). The beads were then equilibrated with RSB-150 buffer containing 0.02% NP-40, and isolated RNA-protein complexes were eluted with sample buffer. Cytoplasmic cell extracts (20 μg) from HeLa cells or immunoprecipitated proteins were separated by electrophoresis on 12% SDS–PAGE gels and transferred to nitrocellulose membranes. For RNase treatment, RNase A/T1 mixture (0.02 mg/ml RNase A and 50 U/ml RNase T1; ThermoFisher) was added to the same cytoplasmic extracts and incubated on ice for 30 min prior to immunoprecipitation. Quantitative Western blotting was performed following the manufacturer's protocol. The membrane was scanned, and the intensity of the protein bands was analyzed on an Odyssey infrared imaging system.

### *In vitro* RNA affinity pull-down assays

Cytoplasmic extracts (2 mg/ml) from HeLa cells with control or target protein knockdown were incubated with 10 nM of biotinylated snRNA in reconstitution buffer containing 0.25 mg/ml *E. coli* tRNA and 1.0 U/μl RNase inhibitor in 96-well plates for 1 h at 4 °C, with gentle mixing at 650 rpm. M280 Streptavidin Dynabeads (Invitrogen) were added in 100 μl of RSB-150 buffer containing 0.02% Triton X-100, a protease inhibitor tablet (Roche), and 0.2 U/μl RNase inhibitor to the reaction mixture and incubated for an additional hour. The beads were washed five times in RSB-200 buffer containing 0.02% Triton X-100 using a magnetic bead processor. Bound proteins were eluted by boiling in 10 μl of sample buffer and then analyzed by SDS–PAGE and western blotting.

### Isolation of Gemin5:snRNA complexes and nascent snRNPs in cells

Gemin5-associated snRNAs were isolated by immunopurification using anti-Gemin5 antibodies from HeLa cell extracts (2mg/ml) lysed in RSB-300 buffer, containing 1% (w/v) Empigen BB and 0.5% (w/v) NP-40 for immunoprecipitation. Collected beads were then incubated with 1 mg/ml of final concentration protease K in a buffer [50 mM Tris-HCl (pH 7.5), 150 mM NaCl, 1% SDS, 5 mM EDTA (pH 8.0)] at 30 °C for 30 min. The snRNAs were recovered by phenol: chloroform extraction, DNAse treatment (0.04 U/μl, Ambion), ethanol precipitation, and resuspension in 20 μl of RNase-free DEPC water. The concentrations of snRNAs were determined by UV absorbance at 260 nm. Total snRNPs were isolated by immunopurification using anti-Sm antibodies from HeLa cell extracts lysed in RSB-500 buffer containing 0.1% NP-40, protease inhibitors, and 0.2 U/μl RNase inhibitor. After 1 h incubation at 4 °C, the beads were washed five times with the lysis buffer using the magnetic bead processor. The same procedures for Gemin5:snRNA complexes purification were applied to isolate snRNAs from the beads.

### Reverse transcription and RT–qPCR measurement of snRNAs

Total RNA was extracted from cells using Trizol (Invitrogen). The specific primers for snRNA, 5S, and 5.8S rRNA were used to generate complementary DNAs (cDNAs) using SuperScript IV Reverse Transcriptase Kit (ThermoFisher). Either 300 ng of total RNA or 1.5 μl of immunopurified RNAs were used as a template in a 20 μl reaction. For each snRNA, 1% (v/v) of the cDNA was used for real-time quantitative PCR (RT-qPCR). PCR reactions (10 μl) were carried out using SYBR Green Master mix (ThermoFisher) and a CFX Duet Real-Time PCR system (Bio-Rad). Each snRNA was measured in triplicate. A plasmid encoding snRNA sequence was used as a standard for absolute quantification of immunoprecipitated snRNAs. Details of all primer sequences are provided in Table S3.

### Statistical analysis

Statistical significance among control and experimental groups was assessed using one-way analysis of variance (ANOVA) followed by Bonferroni *post hoc* test. A *p*-value of < 0.05 was considered statistically significant. All analyses were performed using GraphPad Prism version 10.4.2 (GraphPad Software, https://www.graphpad.com/).

### *In vitro* Sm core assembly and U1C binding assays

The quantitative, high-throughput Sm core assembly assay was performed in accordance with a previously established protocol (30). Briefly, cytoplasmic extracts (2 mg/ml) from HeLa cells were incubated with biotinylated snRNAs (10 nM) in reconstitution buffer containing 2.5 mM ATP, 0.25 mg/ml *E coli* tRNA, and 1.0 U/μl RNase inhibitor for 1 h at 30 °C. For competition experiments, biotinylated WT U1 or U2 snRNAs (10 nM) were incubated with increasing concentrations of non-biotinylated pre-U1 mutants (2.5, 5.0, 10.0, and 20.0 nM) in HeLa cytoplasmic extracts for 1 h at 30 °C. Sm core assembled snRNPs were captured using anti-Sm antibodies-immobilized protein G magnetic beads in RSB-500 buffer containing 0.1% NP-40, protease inhibitor, 2 mg/ml heparin sulfate, and 0.2 U/μl RNase inhibitor for an additional hour at 30 °C. The beads were washed four times with RSB-500 buffer containing 0.1% NP-40, followed by a final wash with RSB-150 buffer containing 0.02% NP-40, using a magnetic particle processor. The snRNPs on the beads were incubated in 0.04 μg/ml of horseradish peroxidase–conjugated Avidin in RSB-150 buffer containing 0.02% NP-40 and incubated for 1 h at 30 °C. The beads were then washed as before and resuspended in 150 μl of SuperSignal ELISA Femto substrate (Pierce). Chemiluminescence signals were detected at 495 nm with a BioTek Synergy HTX microplate reader.

For U1C binding assays with U1 snRNAs, a modified Sm core assembly assay was used, with U1C antibody-immobilized beads substituting for anti-Sm antibodies in the procedure. Additionally, *in vitro* Sm core assembled U1 snRNPs were purified in RSB-50 containing 0.02% TritonX-100.

### *In vitro* U1C peptide-binding assays

Biotinylated U1C peptides (0.5 nmol) were first immobilized on Dynabeads MyOne Streptavidin C1 (Invitrogen) in 100 μl of RSB-200 containing 0.02% Triton X-100 for 30 min. The beads were then incubated with HeLa cytoplasmic extracts (2 mg/ml) for 1 h at 4 °C. After incubation, the beads were washed five times with the same buffer using a magnetic particle processor. Bound proteins were eluted by boiling in 25 μl of sample buffer and then analyzed by SDS–PAGE and western blotting.

## Data availability

The authors confirm that the data supporting the findings of this study are available within the article and its supporting information.

## Supporting information

This article contains supporting information.

## Conflict of interest

The authors declare that they have no conflicts of interest with the contents of this article.
