# Supplementary material for: The U1 snRNP-specific protein U1C is a key regulator of SMN complex–mediated snRNP formation
Source: J Biol Chem. 2025 Jul 22;301(9):110514. doi: 10.1016/j.jbc.2025.110514 (PMC12390936; doi:10.1016/j.jbc.2025.110514)
Supplement: Supporting information [file mmc1.pdf]

Supporting information

The U1 snRNP-specific protein U1C is a key regulator of SMN complex–mediated snRNP formation

Duc Minh Ngu[1,†], Sanat Myti[1,†], Ayesha Ali Khan[1,†], Jeanne Keita[1], Tessa Moore[1], Paul Andega[1], Alaa Aziz[1], Ritu Raj[2], Kayunta Johnson-Winters[1], Eul Hyun Suh[2], and Byung Ran So[1,*]

[1]Department of Chemistry and Biochemistry, University of Texas at Arlington, Arlington, TX 76019; [2]Department of Pharmaceutical Sciences, University of North Texas Health Science Center, Fort Worth, TX 76107

† Equal contributions

* Corresponding author

Tel: 817-272-9865

Email: byungran.so@uta.edu

Table S1. U1C C-terminus peptides used for in vitro binding assays in this study.

**U1C Peptide**	**Sequence (5' to 3')**
Without modification	Biotin- GPPMMRPPARPMMVPTRPGMTRPDR -OH
asymmetrically di-methylated arginines (aDMA)	Biotin- GPPMM-R(Me)2AS-PPA-R(Me)2AS-PMMVPT-R(Me)2AS-PGMT-R(Me)2AS-PDR -OH
symmetrically di-methylated arginines (sDMA)	Biotin- GPPMM-R(Me)2S-PPA-R(Me)2S-PMMVPT-R(Me)2S-PGMT-R(Me)2S-PDR -OH

Table S2. Primers used for snRNA plasmid construction and site-directed mutagenesis in this study.

**Target**	**Forward primer (5' to 3')**	**Reverse primer (5' to 3')**
Pre-U2 snRNA	GAA TTC TAA TAC GAC TCA CTA TAG GGA TCG CTT CTC GGC CTT TTG G	CCC AAG CTT ATT TAG GTG ACA CTA TAG AAG GTC TCA ATC CCC GGA GGG GGT GC
Pre-U4snRNA	GAA TTC TAA TAC GAC TCA CTA TAG GGA GCT TTG CGC AGT GGC AGT A	CCC AAG CTT ATT TAG GTG ACA CTA TAG AAG GTC TCG AAA ATT CAG TCT CCG TAG AG
Pre-U5snRNA	GAA TTC TAA TAC GAC TCA CTA TAG GGA TAC TCT GGT TTC TCT TCA GAT	CCC AAG CTT ATT TAG GTG ACA CTA TAG AAG GTC TCA TAT TGT TGG ATT ACC ACA TAT AG
Pre-U1 snRNA^{A3C}	GAC TCA CTA TAG GGA TCC TTA CCT GGC AGG GG	CCC CTG CCA GGT AAG GAT CCC TAT AGT GAG TC
Pre-U1 snRNA^{C25T}	CTG GCA GGG GAG ATA CTA TGA TCA CGA AGG TG	CA CCT TCG TGA TCA TAG TAT CTC CCC TGC CAG
Pre-U1 snRNA^{C46T}	GAT CAC GAA GGT GGT TTT TCC AGG GCG AGG CTT ATC	GAT AAG CCT CGC CCT GGA AAA ACC ACC TTC GTG ATC
Pre-U1 snRNA^{C69T}	GAG GCT TAT CCA TTG TAC TCC GGA TGT GCT G	CAG CAC ATC CGG AGT ACA ATG GAT AAG CCT C
Pre-U1 snRNA^{C144T}	CAT AAT TTG TGG TAG TGG GGG ATT GCG TTC	CGA ACG CAA TCC CCC ACT ACC ACA AAT TAT G
Pre-U2 snRNA (ΔSm)	GAC AAT ATA TTA AAT GGG ATC TGG AGC AGG GAG ATG G	CCA TCT CCC TGC TCC AGA TCC CAT TTA ATA TAT TGT C
Pre-U4 snRNA (ΔSm)	GTC GGC ACT GGC AGA TCT GAC AGT CTC TAC	GTA GAG ACT GTC AGA TCT GCC AGT GCC GAC
Pre-U5 snRNA (ΔSm)	CTG AGT CTT AAC CCA GAT CTG AGG CCT TGC TTT G	CAA AGC AAG GCC TCA GAT CTG GGT TAA GAC TCA G
pBlueScript U1 snRNA^{A3C}	CCC GAA GAT CTC ATC CTT ACC TGG CAG GGG	CCC CTG CCA GGT AAG GAT GAG ATC TTC GGG

Table S3. Primers used for quantitative RT-PCR.

Target	Forward Primer (5' to 3')	Reverse Primer (5' to 3')
U1 snRNA	GAT ACC ATG ATC ACG AAG GTG GTT	CAC AAA TTA TGC AGT CGA GTT TCC
U2 snRNA	ATC GCT TCT CGG CCT TTT	ATT CCA TCT CCC TGC TCC A
U4 snRNA	GCG CGA TTA TTG CTA ATT GAA A	GTC AAA AAT TGC CAG TGC C
U5 snRNA	GGT TTC TCT TCA GAT CGC ATA AAT C	CTC AAA AAA TTG GGT TAA GAC TCA GA
5S rRNA	CGG CCA TAC CAC CCT GAA C	GCG GTC TCC CAT CCA AGT AC
5.8S rRNA	CGG CTC GTG CGT CGA T	CCG CAA GTG CGT TCG AA

Figure legends

Figure S1. Sm site mutant (ΔSm) pre-snRNAs show defective Sm core assembly compared to wild-type snRNAs. Sm core assembly activity in HeLa cells was measured for each pre-snRNA with an Sm site mutant (ΔSm) and compared to wild-type (WT) snRNA, which was set at 100% activity. Error bars represent the standard deviation (SD) from three biological replicates.

Figure S2. Overexpression of U1 snRNP-specific proteins leads to differential Sm core assembly across all snRNAs. (**A**) Western blot analysis of overexpressed U1 snRNP-specific proteins (myc–U1-70K, myc–U1A, and myc–U1C) compared to endogenous proteins (set at 100%). Input lanes show 10 μg of each of cytoplasmic HEK293T cell extracts. Sm B/B' represents Sm proteins and tubulin is used as the loading control. (**B**) Sm core assembly activity for each snRNA was compared to control extracts (100% activity). Error bars represent the SD from three biological replicates.

Figure S3. SMN complex proteins associate with U1 snRNP-specific proteins. (A) Co-immunoprecipitation of SMN and Gemin5 was performed using target-specific antibodies, followed by SDS-PAGE and Western blot analysis with antibodies against SMN complex components and U1 snRNP-specific proteins. Tubulin was used as a cytoplasmic loading control. (B) U1-70K and U1C were immunoprecipitated from RNase-treated cytoplasmic lysates of HeLa cells, and the precipitates were analyzed by Western blotting using antibodies against SMN complex and U1 snRNP-specific proteins. Magoh and hnRNPC were included as cytoplasmic and nuclear marker proteins, respectively. The input lane represents 2% of the total cytoplasmic extract used for immunoprecipitation. The IgG control lane shows background signal from immunoprecipitation with mouse immunoglobulin G.

Figure S4. The 5'-end mutations in U1 snRNA reduce Sm core assembly compared to wild-type. The Sm core assembly activity of U1 snRNA mutants (C4G, A7G, C9T, T10A, and G12A) was measured and compared to wild-type. Error bars represent the SD from three biological replicates.

Figure S5. U1 snRNA mutants retain the interaction with SMN, Gemin5 or U1-70K proteins. WB analysis of SMN complex proteins bound to biotinylated pre-U1 (WT and point mutants) in cytoplasmic HeLa cells in the presence of ATP. The input lanes show 10% of the cell extracts used.

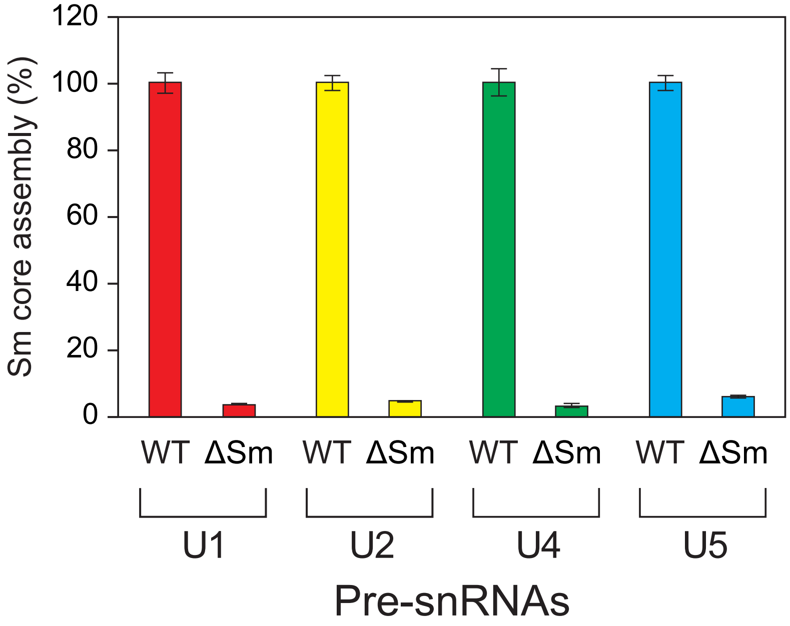
Sm core assembly (%)
120
100
80
60
40
20
0
WT ΔSm
WT ΔSm
WT ΔSm
WT ΔSm
U1
U2
U4
U5
Pre-snRNAs

Figure S1

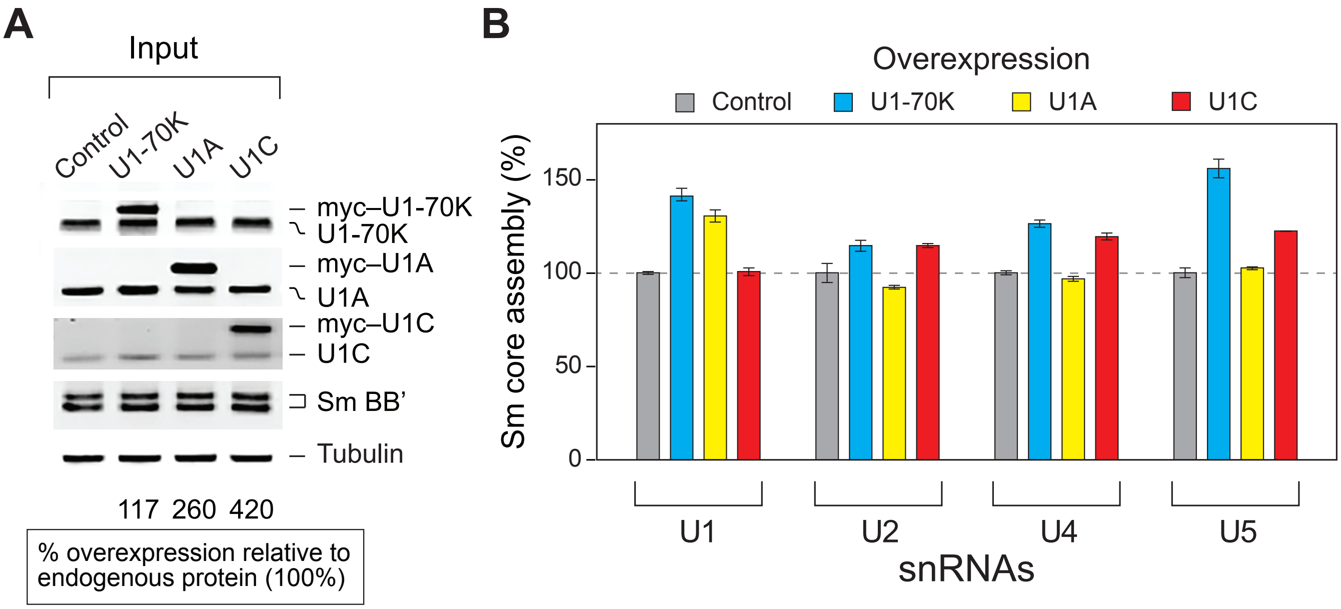
A
Input
Control
U1-70K
U1A
U1C
myc–U1-70K
U1-70K
myc–U1A
U1A
myc–U1C
U1C
Sm BB'
Tubulin
117 260 420
% overexpression relative to endogenous protein (100%)
B
Overexpression
Control
U1-70K
U1A
U1C
Sm core assembly (%)
150
100
50
0
U1
U2
U4
U5
snRNAs

Figure S2

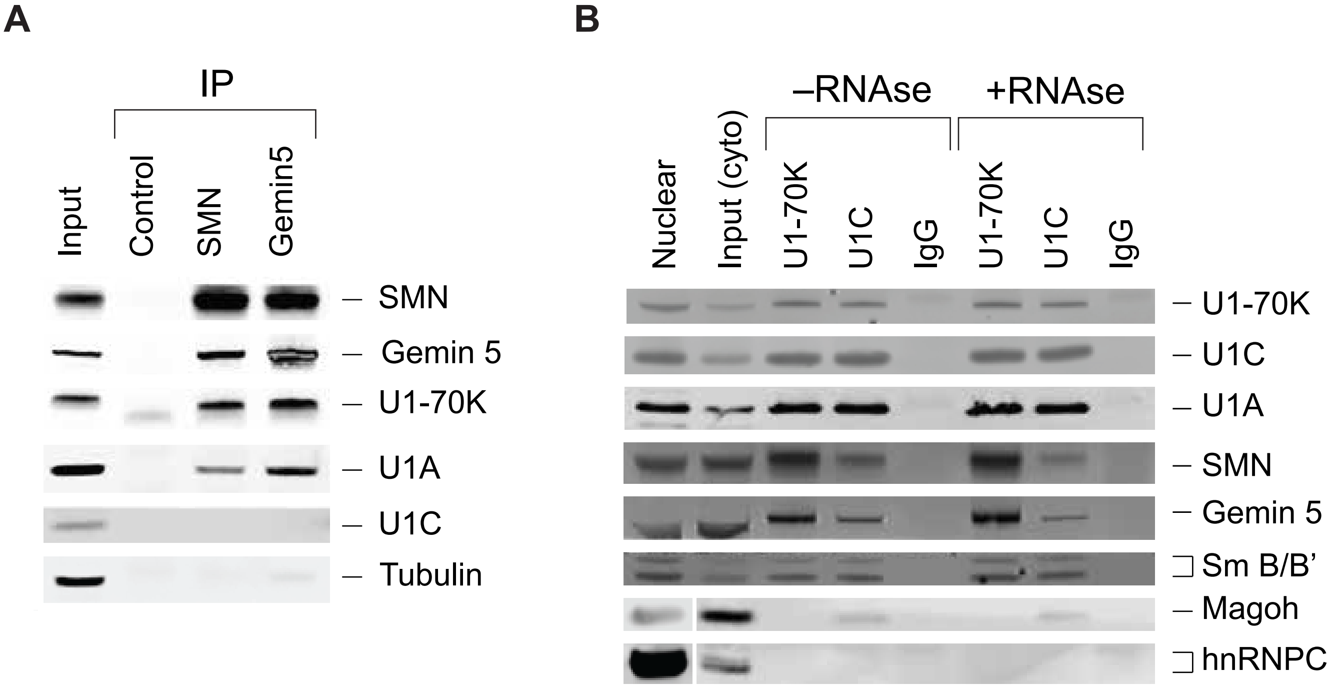
A
IP
Input
Control
SMN
Gemin5
SMN
Gemin 5
U1-70K
U1A
U1C
Tubulin
B
–RNAse
+RNAse
Nuclear
Input (cyto)
U1-70K
U1C
IgG
U1-70K
U1C
IgG
U1-70K
U1C
U1A
SMN
Gemin 5
Sm B/B’
Magoh
hnRNPC

Figure S3

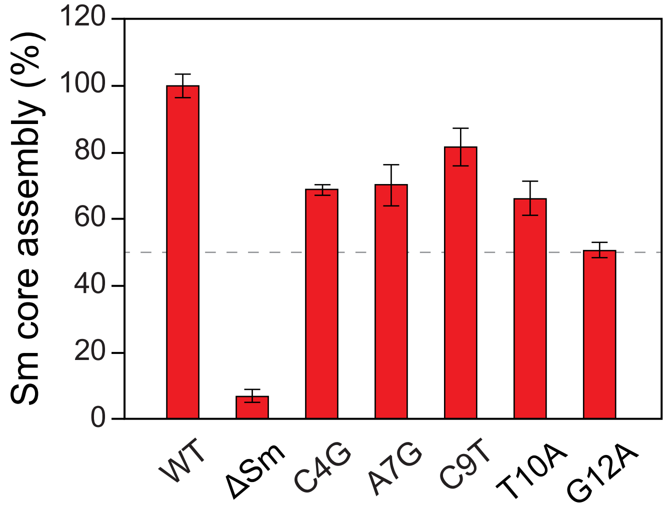
Sm core assembly (%)
120
100
80
60
40
20
0
WT
ΔSm
C4G
A7G
C9T
T10A
G12A

Figure S4

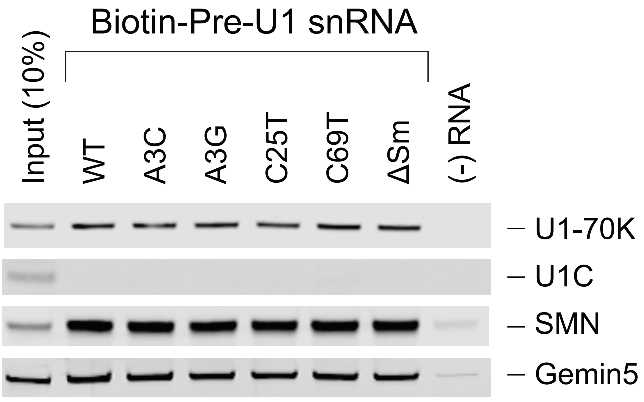
Biotin-Pre-U1 snRNA
Input (10%)
WT
A3C
A3G
C25T
C69T
ΔSm
(-) RNA
U1-70K
U1C
SMN
Gemin5

Figure S5
